# Machine Learning and Deep Learning in Spinal Injury: A Narrative Review of Algorithms in Diagnosis and Prognosis

**DOI:** 10.3390/jcm13030705

**Published:** 2024-01-25

**Authors:** Satoshi Maki, Takeo Furuya, Masahiro Inoue, Yasuhiro Shiga, Kazuhide Inage, Yawara Eguchi, Sumihisa Orita, Seiji Ohtori

**Affiliations:** 1 Department of Orthopaedic Surgery, Graduate School of Medicine, Chiba University, Chiba 260-8670, Japan; 2 Center for Frontier Medical Engineering, Chiba University, Chiba 263-8522, Japan

**Keywords:** spinal injuries, cervical fractures, thoracolumbar fractures, machine learning, deep learning

## Abstract

Spinal injuries, including cervical and thoracolumbar fractures, continue to be a major public health concern. Recent advancements in machine learning and deep learning technologies offer exciting prospects for improving both diagnostic and prognostic approaches in spinal injury care. This narrative review systematically explores the practical utility of these computational methods, with a focus on their application in imaging techniques such as computed tomography (CT) and magnetic resonance imaging (MRI), as well as in structured clinical data. Of the 39 studies included, 34 were focused on diagnostic applications, chiefly using deep learning to carry out tasks like vertebral fracture identification, differentiation between benign and malignant fractures, and AO fracture classification. The remaining five were prognostic, using machine learning to analyze parameters for predicting outcomes such as vertebral collapse and future fracture risk. This review highlights the potential benefit of machine learning and deep learning in spinal injury care, especially their roles in enhancing diagnostic capabilities, detailed fracture characterization, risk assessments, and individualized treatment planning.

## 1. Introduction

Spinal injuries, encompassing both cervical and thoracolumbar regions, present a significant health concern. Traumatic cervical spine fractures have an incidence rate ranging from 4 to 17 per 100,000 person years in Western populations [1]. Historically, these fractures were more prevalent in younger males due to high-energy traumas like road accidents. However, recent trends show a shift toward elderly individuals, both male and female, sustaining these injuries from common low-energy traumas, such as falls [1]. Thoracolumbar fractures, including osteoporotic vertebral fractures (OVFs), are another key area of focus. The rate of thoracolumbar fractures in blunt trauma patients is 6.9%, with motor vehicle collisions and high-energy falls being the leading causes [2]. OVFs are particularly prevalent in the elderly, often resulting from osteoporosis and caused by low-energy traumas like falls from standing height or less. These fractures can lead to various morbidities, including back pain and a decline in the quality of life [3]. Treatment is primarily conservative but may require surgical intervention in severe cases. Despite the high prevalence of OVFs, osteoporosis is often undertreated, emphasizing the need for improved management and prevention strategies. Given the significant impact on patients’ quality of life and broader societal implications, ensuring accurate and timely diagnosis and prognosis of spinal injuries is crucial for effective treatment without complications. This is especially crucial in an aging society where the prevalence of such injuries, including OVFs, is increasing.

**Figure 1:**
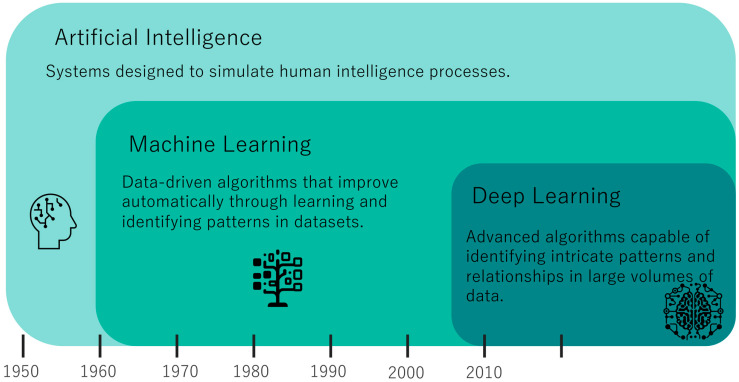
Diagram illustrating the relationship and distinctions between artificial intelligence, machine learning, and deep learning.

**Figure 2:**
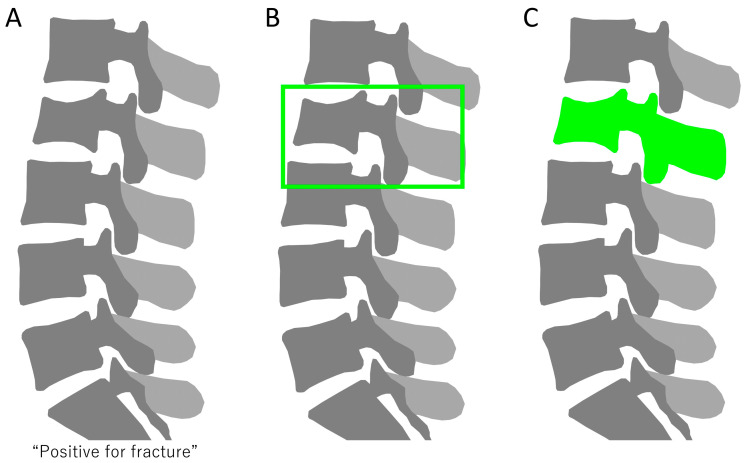
(**A**) Classification of vertebral fractures, identifying the presence or absence of fractures in radiographs. (**B**) Object detection in spine radiographs, with bounding boxes indicating fracture locations. (**C**) Segmentation of vertebral fractures, delineating fractured areas.

Artificial intelligence (AI) is the broadest term, encompassing the creation of machines or software that can perform tasks requiring human-like intelligence. This includes reasoning, learning, problem solving, perception, and language understanding. Machine learning is a subset of AI and involves training algorithms to learn from and make predictions or decisions based on data. It is often used with structured data, such as tables in databases, where the algorithm learns to identify patterns and relationships. The basic principle is to feed data into an algorithm, allowing it to learn from that data, and then use the learned model to make predictions or decisions on new, unseen data. Deep learning is a subset of machine learning that uses neural networks with many layers to analyze complex patterns in data (Figure 1). It is particularly effective for the following: classification problems: assigning data into predefined categories, like diagnosing diseases from medical images; object detection: identifying and locating objects within images, vital for applications like medical imaging to pinpoint abnormalities; and segmentation: dividing an image into segments to identify and locate different parts, such as different tissues in a medical scan (Figure 2). Machine learning and deep learning, both integral components of artificial intelligence (AI), are more than just trending topics; they are technologies that bring about significant change and have begun to bring about major changes in multiple areas, including healthcare [4]. In spine care, advancements in computational methods, especially machine learning and deep learning, have demonstrated significant potential in enhancing the diagnosis and prognosis of spine lesions [5,6,7,8,9]. These technologies have been particularly effective in analyzing various types of data, including imaging data from CT scans and MRI, as well as structured clinical data. Machine learning and deep learning have not only shown promise in improving surgical planning, patient selection, and postoperative care but also in driving personalized medicine approaches [5,6,7,8,9]. The integration of machine learning and deep learning in spine care holds the potential to elevate patient outcomes and reduce healthcare costs. Notably, while there exist several studies that review the application of machine learning and deep learning in spinal surgeries and spinal cord injuries (SCIs), relatively little focus has been given to their role in spinal injuries [5,6,7,8,9]. Questions regarding their robustness and generalizability in spinal injury care across different settings remain largely unexplored. This lack of comprehensive understanding motivates the need for a narrative review that explores both the diagnostic and prognostic aspects of machine learning and deep learning in spinal injury.

The purpose of this narrative review is to explore the scope and clinical applicability of machine learning and deep learning algorithms in the diagnosis and prognosis of spinal injuries. We aim to provide a comprehensive overview of the existing literature, focusing on studies that utilize either imaging or tabulated data, to offer valuable insights that could guide future research and clinical practice in the diagnosis and treatment of spinal injury.

## 2. Materials and Methods

### 2.1. Search Criteria

We searched the PubMed database using the terms (“Cervical Fracture” OR “Thoracic Fracture” OR “Lumbar Fracture” OR “Spinal Fracture” OR “Spine Fracture” OR “Vertebral Fractures” OR “Spinal Trauma” OR “Vertebral Injury” OR “Fracture Screening”) AND (“Artificial Intelligence” OR “Machine Learning” OR “Deep Learning” OR “Neural Network” OR “Computer Vision” OR “Automated”) AND (“2013/01/01”[Date-Publication]: “2023/09/11”[Date-Publication]). Studies published in English from 1 January 2013 to 11 September 2023 were considered. The starting point of 2013 was specifically chosen to include research that utilizes modern deep learning techniques. No limitations were set on the geographical locations of the studies.

### 2.2. Eligibility Criteria

To be included in the review, studies needed to implement machine learning or deep learning algorithms, focus on the diagnosis or prognosis of spinal injuries, and utilize either imaging or tabulated clinical data. On the other hand, we excluded studies that specifically focused on spinal cord injuries, those that aimed to estimate fracture risk based on bone density, as well as reviews and systematic reviews. Studies published in languages other than English, case studies, and studies without full text available were also excluded.

### 2.3. Study Selection

The initial search yielded 86 papers. Titles and abstracts were initially screened for relevance by the first author (SM) of this review. In cases where eligibility was uncertain, two independent reviewers (TF and MI) were consulted. Any disagreement between the reviewers and the first author was resolved through discussion.

### 2.4. Review Framework and Components

Our narrative review was organized to comprehensively cover the application of machine learning and deep learning in the diagnosis and prognosis of spinal injuries. To ensure a full scope of information, we focused on the following components:

Diagnosis/Prognosis: The role the algorithms played in diagnosing or predicting the outcome of the spinal injuries.
Target Pathology: The specific type of spinal injury being investigated in each study.
Patients Studied: Number of the patients who participated in the studies.
Images Studied: Number of images analyzed in the studies.
Type of Data: Categories of data, such as imaging or tabulated clinical data, used in the study.
Computational Task: The specific computational goal for which machine learning or deep learning algorithms were employed, such as object detection, segmentation, or classification.
Machine Learning Models: Types of machine learning and deep learning algorithms used, such as decision trees, random forests, etc.
Summaries: Summaries of the key findings and essential takeaways from each individual study.

## 3. Results

**Figure 3:**
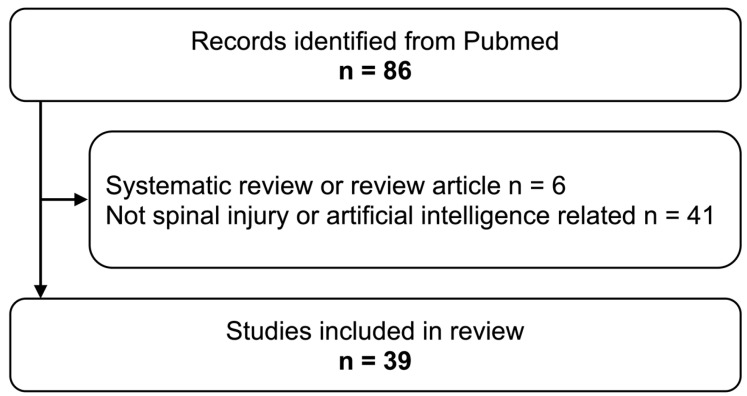
Flowchart of study selection criteria for the review.

Out of the initial 86 papers identified, 39 met the inclusion criteria and were included in the final analysis (Figure 3). These studies were divided into two main categories: those focusing on diagnostic algorithms (34 articles) (Table 1) and those dedicated to prognostic evaluations (5 articles) (Table 2).

### 3.1. Algorithms for Diagnosing Spinal Injuries

The majority of the 34 diagnostic studies employed deep learning algorithms using imaging modalities such as CT, MRI, DXA, and X-ray. The algorithms used in these studies were mainly convolutional neural networks. 

### 3.2. Algorithms for Predicting Spinal Injury Outcomes

In contrast, four out of the five prognostic studies used machine learning algorithms such as the decision tree model, random forest, and XGBoost, relying on tabulated clinical data. These prognostic studies covered topics such as the progression of vertebral collapse, imminent new vertebral fractures after vertebral augmentation, future vertebral fractures, treatment-related risk factors, and predictions of nonunion. Much like the diagnostic studies, the majority of these prognostic studies also focused on thoracolumbar injuries.

### 3.3. Types of Data and Study Design

Both imaging data and tabulated clinical data were employed across the selected studies, though each type of data was more prevalent in one of the two main focus areas—imaging in diagnosis and tabulated data in prognosis. These studies predominantly targeted thoracolumbar injuries, with only five focusing on cervical spinal injuries. The retrospective design was dominant in both categories, accounting for 37 of the 39 studies, indicating a gap in prospective research in this field.

## 4. Discussion

### 4.1. Overview of Machine Learning Applications in Spinal Care

In this review, we explored a diverse range of objectives in the studies reviewed, encompassing everything from the diagnostic precision in morphometric vertebral fracture analysis to the prediction of imminent and future vertebral fractures. This range highlights the expansive yet focused applications of machine learning and deep learning in spinal injury care. Deep learning techniques, especially CNNs, have been at the forefront in refining diagnostic approaches. These include enhancing the accuracy in the detection and classification of fractures, as well as in the critical task of differentiating between benign and malignant fractures using complex imaging data. On the other hand, machine learning methods, such as random forests and SVMs, have primarily been applied for prognostic purposes, offering valuable insights into the progression of vertebral collapse and identifying treatment-related risk factors using structured clinical data. While prognostic studies utilizing machine learning are fewer, they provide significant contributions to understanding spinal injury outcomes. This distinction highlights the tailored application of each technology to the unique challenges presented by spinal injury diagnosis and prognosis. The diversity and complexity of spinal injuries, along with their varied treatments, underscore the utility of machine learning and deep learning in facilitating more individualized care approaches.

### 4.2. Diagnostic Approaches

#### 4.2.1. X-ray 

##### Diagnostic Accuracy Using Entire X-rays

The accuracy of determining the presence of vertebral fractures using plain radiographs of the entire thoracolumbar spine can be challenging because identifying vertebral fractures in a broad region of interest might be a complex task for neural networks. A study by Chen et al. using a CNN reported an accuracy of around 73.59% for vertebral fracture identification with these X-ray images [15]. Murata et al. trained a CNN to identify thoracolumbar vertebral fractures using X-ray images and achieved accuracy, sensitivity, and specificity rates of 86.0%, 84.7%, and 87.3%, respectively [41]. 

##### Evaluation of Manually Cropped Regions

Rosenberg et al. focused on using deep learning models to diagnose traumatic thoracolumbar fractures by analyzing individual vertebra regions that were manually cropped from the lateral radiographs, finding that the ResNet18 architecture outperformed VGG16 with higher sensitivity (91%), specificity (89%), and accuracy (88%) [22].

##### Object Detection and Ensemble Approaches

An alternative and potentially more accurate approach may involve first using object detection algorithms to identify individual vertebrae, followed by classification or segmentation to assess their condition [16,42]. Li et al. used a deep learning ensemble on 941 lateral spine radiographs to detect osteoporotic lumbar vertebral fractures, achieving 93% accuracy, 91% sensitivity, and 93% specificity [42]. Chou et al. employed an ensemble model on 1305 thoracolumbar X-rays to identify vertebral fractures, attaining similar performance metrics—93.36% accuracy, 88.97% sensitivity, and 94.26% specificity [16]. Shen et al. developed and validated a deep-learning-based system (AI_OVF_SH) for diagnosing and grading osteoporotic vertebral fractures using plain radiographs [25]. In external validation, the system demonstrated high performance, achieving an accuracy of 96.85%, sensitivity of 83.35%, and specificity of 94.70% for detecting all types of fractures [25].

##### Cervical Spine 

Naguib et al. developed a computer-aided diagnosis system based on deep learning algorithms, specifically AlexNet and GoogleNet, to classify cervical spine injuries as either fractures or dislocations using X-ray images [18].

##### Age-Specific Algorithm Performance

Alqahtani et al. reported that a software program initially trained on adult spinal fracture data, using DXA and X-ray for diagnosis, showed poor sensitivity and specificity when applied to pediatric cases. This underscores the need for algorithms to consider the age of the patient for the target condition in their training data [10,11,12]. 

#### 4.2.2. DEXA

Mehta et al. demonstrated that using ancillary numerical data obtained from routine DEXA scans, support vector machine (SVM) analysis can accurately identify lumbar spine fractures. The method, specifically with a linear kernel SVM, yielded a high area under the curve (AUC = 0.9258), indicating that it can detect fractures without the need for additional imaging or radiation exposure [43]. Derkatch et al. found that CNNs, specifically InceptionResNetV2 and DenseNet, can accurately identify vertebral fractures in thoracolumbar regions using images from DXA scans. The CNN ensemble achieved an area under the receiver operating characteristic curve of 0.94, with a sensitivity of 87.4% and a specificity of 88.4% [17]. Monchka et al. showed that CNNs can accurately identify vertebral fractures in images from multiple types of DXA scanners [44]. The final CNN ensemble model achieved a sensitivity of 91.9%, a specificity of 99.0%, and an F1-score of 86.1% [44]. The F1-score is the harmonic mean of precision and recall, providing a balance between the two.

#### 4.2.3. CT

##### Automated Detection Algorithms

Roux et al.’s study employed software from Zebra Medical Vision to perform opportunistic screening for vertebral fractures and osteoporosis in more than 150,000 routine lumbar spine CT scans, achieving success rates of 82% for vertebral fracture assessment and 87% for Hounsfield Unit (HU) measurements, showing the potential to enhance diagnostic accuracy in large-scale screening [23]. Tomita et al. utilized deep neural networks for the automatic detection of osteoporotic vertebral fractures in CT scans, achieving an accuracy of 89.2% and an F1-score of 90.8% [27]. The study by Inoue et al. used Faster R-CNN to detect fractures in the pelvis, ribs, and spine, offering a comprehensive approach for trauma cases [45]. However, the model’s sensitivity is lower compared to specialized spinal fracture models, suggesting room for improvement. Rueckel et al. investigated the utility of AI assistance in detecting missed thoracic findings, including vertebral fractures, in emergency whole-body CT scans [24]. The study found that 57.8% of suspicious vertebral bodies were identified solely by the AI, while radiologists alone detected 29.7%, and both AI and radiologists detected 12.5%, suggesting that AI assistance can significantly reduce the rate of missed thoracic findings in emergency settings [24].

##### Fracture Classification

Some models not only diagnose the presence of fractures but also classify the type of fracture. For example, Chen et al. used deep learning for high-accuracy AO (Arbeitsgemeinschaft für Osteosynthesefragen) classification of thoracolumbar fractures [14]. On the other hand, Doerr et al. developed a model to categorize vertebral morphology and determine posterior ligamentous complex integrity for the purpose of assigning Thoracolumbar Injury Classification and Severity Score (TLICS), both using CT scans [46]. Zhang et al. developed a multistage system using CNNs (U-net, GCN, 3D-ResNet) that can automatically detect and classify acute thoracolumbar vertebral body fractures on CT images with high-accuracy AO classification—achieving a sensitivity of 95.23%, an overall accuracy of 97.93%, a specificity of 98.35%, and balanced accuracy rates ranging from 79.56% to 94.5% for different fracture types according to AO classification [34].

##### Opportunistic Screening and Fracture Liaison

The ability to screen for vertebral fractures from CT scans taken for other purposes highlights the strength of AI in this context. Nicolaes et al. used a 3D CNN to identify thoracolumbar vertebral fractures from CT scans with 94% sensitivity and 93% specificity. The algorithm showed an AUC of 0.94, making it highly effective in opportunistically identifying vertebral fractures in routine CT scans [19]. Ong et al. evaluated the efficacy of a machine learning algorithm in identifying vertebral fractures from CT scans, revealing that the algorithm detected fractures in 19.1% of 4461 patients, outperforming hospital radiologists whose reports only mentioned 49% of these fractures [20]. Valentinitsch et al. used a random forest classifier and 3D texture features for opportunistic osteoporosis screening in thoracolumbar spine multi-detector CT scans, demonstrating high discriminatory power (AUC = 0.88) and outperforming global vertebral bone mineral density (vBMD) alone in identifying vertebral fractures [28].

##### 3D-Based Algorithms

While 3D volume data may have higher computational costs, they hold the potential for a more accurate assessment of fracture morphology. Burns et al. used 3D volume data from CT scans to achieve high sensitivity and low false-positive rates in detecting and anatomically localizing thoracic and lumbar vertebral body fractures [13]. Zakharov et al. developed an anchor-free vertebra detection network using convolutional neural networks that effectively localizes the vertebral column in 3D CT images and simultaneously detects individual vertebrae and quantifies fractures in 2D, demonstrating strong performance with an AUC of 0.95, sensitivity of 0.85, and specificity of 0.9 on the challenging VerSe dataset containing various unseen vertebra fracture types [33]. Zhang et al. introduced a novel multi-scale attention-guided network (MAGNet) for diagnosing thoracolumbar vertebral fractures and three-column injuries, achieving an AUC of 0.884 for vertebral fracture diagnosis and 0.920 for three-column injury diagnosis, both with high precision on CT images [35].

##### Distinguishing Benign from Malignant Vertebral Fractures

Differentiating between osteoporotic and malignant vertebral fractures is crucial, especially among elderly patients, for appropriate treatment planning and prognosis. Goller et al. in 2023 used a CNN-based framework to distinguish between benign and malignant thoracolumbar vertebral fractures using CT-based texture features. Their study found statistically significant differences in these features between benign and malignant fractures [47]. Park et al. developed an automated segmentation algorithm for CT scans of thoracolumbar fractures, showing that its performance in predicting fracture malignancy was comparable to human expert segmentation [21].

##### Cervical Spine

Research on detecting cervical fractures is comparatively less abundant than studies on thoracolumbar fractures. Golla et al. in 2023 used convolutional neural networks to detect cervical spine fractures from CT scans. Their algorithm detected 87.2% of fractures with an average of 3.5 false positives per case, using a spinal canal aligned volumes of interest (VOI) approach [48]. Small et al. evaluated a CNN developed by Aidoc for detecting cervical spine fractures using CT scans, reporting a 92% accuracy rate with 76% sensitivity and 97% specificity [26]. Voter et al. evaluated the diagnostic performance of a deep learning algorithm by Aidoc for detecting cervical spine fractures in CT scans, finding a relatively low sensitivity of 54.9% but a high specificity of 94.1% [29]. 

#### 4.2.4. MRI

##### Diagnosing Fresh Osteoporotic Vertebral Fracture

Yabu et al. used an ensemble method comprising VGG16, VGG19, DenseNet201, and ResNet50 to diagnose fresh osteoporotic vertebral fractures in thoracolumbar MRI scans, finding that the CNN’s performance metrics were comparable to those of two spine surgeons [30].

##### Distinguishing Benign from Malignant Vertebral Fractures

Yoda et al. employed a deep convolutional neural network using Xception architecture to differentiate osteoporotic and malignant vertebral fractures using MRI [32]. The model’s accuracy was statistically equal or superior to that of spine surgeons. Yeh et al. applied a ResNet50 deep learning algorithm to MRI scans of the whole spine for distinguishing between benign and malignant vertebral fractures, achieving an accuracy of 92% and potentially improving diagnostic performance for less experienced clinicians [31].

### 4.3. Prognostic Approaches

Research focused on predicting prognosis is relatively scarce compared to diagnosis and classification. Most of these few studies do not directly predict from images but use machine learning on parameters extracted from images. For instance, Cho et al. worked on predicting the progression of vertebral collapse in osteoporotic vertebral fractures. Using manually extracted parameters from X-ray and MRI images, they employed machine learning techniques like decision trees and random forests [36]. Jiang et al. employed machine learning models, specifically random survival forest and COX proportional hazard analysis, to predict new osteoporotic vertebral compression fractures after vertebral augmentation using T1W MR images [37]. Kong et al. focused on predicting osteoporotic fractures in the lumbosacral spine using X-ray images and deep learning in a longitudinal cohort study [38]. Their DeepSurv model, trained on both images and clinical features, outperformed traditional methods like FRAX and CoxPH in terms of C-index values, suggesting its potential for more accurate fracture prognosis [38]. Takahashi et al. employed machine learning models including logistic regression, decision trees, XGBoost, and RF to improve the prediction of nonunion following osteoporotic vertebral fractures in the thoracolumbar region, using MRI data from 505 patients [40]. The study found high prognostic accuracy with AUC scores of 0.860 and 0.845 for RF and XGBoost, respectively [40]. Leister et al. aimed to identify treatment-related risk factors for nonunion of odontoid fractures in the cervical spine using machine learning models, specifically XGBoost and binary logistic regression [39]. The study found moderate predictive power, with an AUC of 0.68 for the XGBoost model and 0.71 for the binary logistic regression model, suggesting their potential utility in understanding treatment-related risks [39].

### 4.4. Advantages and Disadvantages of Each Model

Machine learning and deep learning methods play a crucial role in diagnosing and predicting spinal injuries, each with its own strengths and drawbacks. Techniques such as CNNs, random forests, SVMs, XGBoost, and recurrent neural networks (RNNs) are important in this field. CNNs are particularly good at recognizing patterns in images, including object detection and segmentation, which is helpful for examining complex data like X-ray, CT, and MRI scans [49,50]. Random forests are strong at analyzing big datasets, SVMs work well in handling data with many features, and XGBoost is known for its execution speed. RNNs are especially useful for data that are ordered over time. These methods have their own limitations regarding data requirements, computing power, and how easy it is to understand their results [51]. For the advantages and disadvantages of each model related to spinal injury care, please see Table 3. Choosing the right method depends on the specific needs of spinal injury data, the computing resources available, and finding a balance between precision and interpretability.

### 4.5. Future Direction

Future research in spinal fracture care should prioritize the development and refinement of predictive algorithms offered by machine learning to enhance both preoperative and postoperative care. The following specific directions are worth considering:

Integration of diverse data for tailored treatment: Emphasis should be placed on integrating clinical and radiological data to tailor treatment plans to individual patient profiles. This approach has the potential to significantly improve diagnosis accuracy and the efficacy of interventions like vertebral augmentation and surgical stabilization [52]. 

Focus on cervical spine fractures: Given the relative underrepresentation of cervical spine fractures in the current literature, future studies should particularly focus on this area. Research could explore new diagnostic techniques and frameworks for treatment decision making.

Application of large language models: The use of large language models in analyzing medical texts, such as patient records and radiology reports, presents a promising avenue for improving diagnosis accuracy and treatment personalization. Future studies should explore the potential of these models, as highlighted in the recent study [53].

Development of multimodal language models: Research should be directed toward developing multimodal models that process and integrate different types of data (imaging, clinical notes, sensor data) for a comprehensive understanding of the patient’s condition [54]. This approach aligns with the personalized and data-driven healthcare trend. 

Collaboration and regulatory compliance: The effective translation of these technologies into clinical practice necessitates collaboration between engineers, clinicians, and regulatory agencies in model validation, auditing, quality monitoring, and updates. Clinical deployment protocols must delineate the role of algorithmic assistance and human clinical judgment, ensuring accountability.

Expanding prognostic research: There is a need to expand research into machine learning algorithms for predicting outcomes like vertebral collapse, future fracture risk, and nonunion. This expansion is crucial for more informed surgical decision making and aligns with ethical and practical considerations for clinical safety and effectiveness.

### 4.6. Limitations

While our review provides a broad overview, it is important to recognize certain inherent limitations that should be addressed in future studies:

Retrospective nature of studies: A significant limitation of our review is the reliance on retrospective studies. Predictive modeling, a key focus of our research tends to yield more robust and generalizable results when based on prospectively collected data. This limitation underscores the need for future studies to prioritize prospective data collection.

Lack of external validation: Another limitation is the absence of external validation in most of the machine learning models discussed. External validation is essential to confirm the models’ reliability, efficacy, and safety in diverse clinical settings.

Explainability in deep learning: The lack of explainability in many deep learning studies presents a barrier to their acceptance and practical utility in clinical settings. Clinicians require transparent and understandable models to make informed decisions.

These limitations not only highlight areas for improvement but also guide future research directions. Prospective studies, rigorous external validation processes, and the development of explainable AI models should be the focus of subsequent investigations in spinal injury care. By addressing these limitations, future research can significantly contribute to advancing the field.

## 5. Conclusions

In conclusion, this review underscores the growing importance of machine learning and deep learning in spinal injury care. These technologies are not just enhancing diagnostic methods for morphometric vertebral fractures but are also widening the scope for near and long-term fracture prediction. Their potential is most apparent in offering more precise diagnoses and differentiating between benign and malignant fractures. Despite being less prevalent, prognostic studies offer important insights, such as vertebral collapse progression and treatment-related risk factors. These computational approaches seem well positioned to manage the complexity and diversity of spinal injuries, suggesting they may play a meaningful role in facilitating more individualized care strategies. As these technologies continue to develop, they hold the potential to make significant contributions to both the scientific understanding and clinical management of spinal injuries. By aligning data-driven analyses with individual patient needs, machine learning and deep learning could become increasingly relevant in the field of spinal injury care.

## Figures and Tables

**Table 1:** List of diagnostic studies selected for final analysis.

Author(s)	Year	Target Pathology	Patients Studied	Images Studied	Type of Data	Task	Machine Learning Models	Summary
Alqahtani et al. [10]	2020	Vertebral fractures in children	420	420	DXA	Point Detection	AVERT	The vertebral fracture analysis technique for adults is not reliable for fractures in children, suggesting the need for a pediatric standard.
Alqahtani et al. [11]	2019	Vertebral fractures in children	100	100	X-ray, DXA	Point Detection	AVERT and SpineAnalyzer	The study found that neither AVERT nor SpineAnalyzer is reliably satisfactory for vertebral fracture diagnosis in children.
Alqahtani et al. [12]	2017	Vertebral fractures in children	137	1781 (vertebrae)	X-ray	Point Detection	SpineAnalyzer	The current software is inadequate for diagnosing vertebral fractures in children and needs retraining to include child-specific factors.
Burns et al. [13]	2016	Thoracolumbar vertebral fractures	104	3D volume data	CT	Object Detection, Segmentation	Support vector machine	A fully automated computer system detects and anatomically localizes vertebral body fractures on CT images.
Chen et al. [14]	2022	Thoracolumbar vertebral fractures (AO classification)	1130	1130	CT	Object Detection	Faster R-CNN	A deep-learning-based classification method achieves high accuracy in classifying thoracolumbar vertebral fractures.
Chen et al. [15]	2021	Osteoporotic vertebral fractures	1306	1306	X-ray	Classification	ResNeXt-50	A deep learning algorithm was developed to detect and visualize vertebral fractures on plain frontal radiographs.
Chou et al. [16]	2022	Thoracolumbar vertebral fractures	1305	1305	X-ray,	Object Detection, Classification, Segmentation	Ensemble model, YOLOv3	The AI model trained on older adult data effectively identified vertebral fractures in older patients and intact vertebrae in younger adults.
Derkatch et al. [17]	2019	Osteoporotic vertebral fractures	12,742	12,742	DXA	Classification	Inception-ResNet V2 and DenseNet	CNNs can identify vertebral fractures on vertebral fracture assessment images with high accuracy and predict clinical fracture outcomes.
Naguib et al. [18]	2023	Cervical spine fracture	NA	2009	X-ray	Classification	AlexNet and GoogLeNet	A computer-aided-diagnosis system based on deep learning for classifying cervical spine injuries as fractures or dislocations.
Nicolaes et al. [19]	2023	Osteoporotic vertebral fractures	4810	3D volume data	CT	Object Detection, Classification	3D CNN (model not specified)	The CNN algorithm achieved 94% sensitivity and 93% specificity in identifying vertebral fractures in routine CT scans.
Ong et al. [20]	2021	Osteoporotic vertebral fractures	850	4461	CT	Point Detection	Model developed by Optasia Medical	Integration of a vertebral fracture identification service into a Fracture Liaison Service is possible and increases workload.
Park et al. [21]	2022	Benign and malignant vertebral fractures	276	529	CT	Segmentation, Classification	U-Net, AsanFEx in MATLAB for radiomics	An algorithm for segmenting fractured vertebrae on CT scans was created, performing on par with experts in predicting fracture malignancy.
Rosenberg et al. [22]	2022	Thoracolumbar vertebral fractures	151	630 (vertebrae)	X-ray	Classification	ResNet18, VGG16	A deep learning model was developed to accurately detect traumatic thoracolumbar fractures on sagittal radiographs.
Roux et al. [23]	2022	Osteoporotic vertebral fractures	152,268	NA	CT	Classification	Software (Zebra Medical Vision)	Opportunistic screening of vertebral fractures and osteoporosis on more than 150,000 routine CT scans.
Rueckel et al. [24]	2021	Missed thoracic findings in emergency CT	105	NA	CT	Segmentation, Point Detection	Software (Siemens)	AI assistance reduces missed thoracic findings in emergency whole-body CT scans.
Shen et al. [25]	2023	Osteoporotic vertebral fractures	12,673	12,673	X-ray	Object Detection, Segmentation	CNN (model not specified)	A deep-learning-based system for diagnosing and grading osteoporotic vertebral fractures on plain radiographs was developed and validated.
Small et al. [26]	2021	Cervical spine fractures	665	NA	CT	Segmentation, Classification	CNN developed by Aidoc	A CNN demonstrated 92% accuracy in detecting cervical spine fractures on CT, with potential for worklist prioritization and assisting radiologists.
Tomita et al. [27]	2018	Osteoporotic vertebral fractures	1432	NA	CT	Classification	ResNet34, LSTM	Automatic detection of osteoporotic vertebral fractures on CT scans using deep neural networks.
Valentinitsch et al. [28]	2019	Osteoporotic vertebral fractures	154	NA	CT	Classification	Random forest	An automatic screening tool using 3D texture and regional vBMD excels over global vBMD in detecting vertebral fractures.
Voter et al. [29]	2021	Cervical spine fractures	1904	NA	CT	Segmentation, Classification	Deep learning algorithm (Aidoc)	An AI system for detecting cervical spine fractures showed low accuracy, questioning its generalizability and practical deployment.
Yabu et al. [30]	2021	Osteoporotic vertebral fractures	814	1624	MRI	Classification	Ensemble model	The performance of the CNN in detecting fresh osteoporotic vertebral fractures using MR images was comparable to that of two spine surgeons.
Yeh et al. [31]	2022	Benign and malignant vertebral fractures	190	NA	MRI	Classification	ResNet50	A ResNet50-based deep learning method enhanced less experienced clinicians’ ability to diagnose vertebral fractures from spine MRI.
Yoda et al. [32]	2022	Benign and malignant vertebral fractures	97	697	MRI	Classification	Xception	A CNN model distinguished osteoporotic and malignant vertebral fractures on MRI with accuracy comparable to spine surgeons.
Zakharov et al. [33]	2023	Osteoporotic vertebral fractures	100	3565	CT	Point Detection	CNN (model not specified)	A two-step algorithm localizes the vertebral column in 3D CT and detects and quantifies individual vertebral fractures in 2D.
Zhang et al. [34]	2023	Thoracolumbar vertebral fractures (AO classification)	1217	NA	CT	Object Detection, Classification	U-Net, GCN, 3D-ResNet	The AO system automatically detects and classifies acute thoracolumbar spine fractures on CT with high accuracy per AO standards.
Zhang et al. [35]	2023	Thoracolumbar vertebral fractures	197	989 (vertebrae)	CT	Object Detection, Classification	Multi-scale attention-guided network	A novel network for diagnosing vertebral fractures and three-column injuries with fracture visualization at a vertebra level.

CT, Computed Tomography; AO, Arbeitsgemeinschaft für Osteosynthesefragen; MRI, Magnetic Resonance Imaging; DXA, Dual-energy X-ray Absorptiometry; CNN, Convolutional Neural Network; LSTM, Long Short-Term Memory; vBMD, Volumetric Bone Mineral Density; GCN, Graph Convolutional Network.

**Table 2:** List of prognostic studies selected for final analysis.

Author(s)	Year	Target Pathology	Patients Studied	Images Studied	Type of Data	Task	Machine Learning Models	Summary
Cho et al. [36]	2023	Osteoporotic vertebral fractures (progression of collapse)	670	NA	X-ray, MRI	Classification	Decision tree, random forest	The model predicts progressive collapse in osteoporotic vertebral fractures using machine learning and statistics.
Jiang et al. [37]	2023	Osteoporotic vertebral fractures (fracture prediction)	235	NA	MRI	Classification	Random survival forest, Cox hazards model	A machine learning model combining radiomics and clinical data predicts new vertebral fractures post-vertebral augmentation.
Kong et al. [38]	2022	Osteoporotic vertebral fractures (fracture prediction)	1595	1595	X-ray	Classification	CNN, DeepSurv	A spine X-ray-based fracture prediction model was developed using deep learning with longitudinal data.
Leister et al. [39]	2023	Odontoid fracture nonunion prediction	415	NA	X-ray, CT	Classification	XGBoost, binary logistic regression	The study aimed to identify treatment-related risk factors for odontoid fracture nonunion using machine learning models.
Takahashi et al. [40]	2022	Osteoporotic vertebral fractures (nonunion prediction)	505	NA	MRI	Classification	Decision tree, XGBoost, random forest	Machine learning models improve nonunion prediction following osteoporotic vertebral fractures.

CT, Computed Tomography; MRI, Magnetic Resonance Imaging; CNN, Convolutional Neural Network.

**Table 3:** Advantages and disadvantages of machine learning models in the context of spinal trauma.

Model	Definition	Advantage	Disadvantage
Support Vector Machines (SVMs)	Supervised learning models used for classification and regression analysis, known for their effectiveness in high-dimensional spaces.	Effective in classifying spinal trauma cases based on imaging features. Good performance with smaller datasets.	Struggle with very large datasets. Less accurate in noisy data environments, like mixed injury types.
Random Forests	An ensemble learning method for classification and regression, using multiple decision trees.	Used for predicting the prognosis of spinal injury. Strong in handling diverse clinical data types without overfitting.	Complexity of the model can hinder its interpretability.
XGBoost	A scalable and efficient version of gradient boosted decision trees.	Applied in spinal trauma for outcome prediction and risk assessment. Known for its quick processing of complex datasets.	Can overfit if not properly tuned.
Convolutional Neural Networks (CNNs)	Deep neural networks particularly effective in analyzing images, used for pattern recognition in images.	Widely used for detecting fractures and assessing injury severity in spinal trauma imaging. Effective in image-based analysis and interpretation.	Require large, diverse datasets for training. Demand high computational resources.
Recurrent Neural Networks (RNNs)	Neural networks designed to process sequential data, recognizing patterns in time-dependent data.	Utilized in tracking and predicting patient recovery progress and response to treatment in spinal trauma cases.	Difficult to train the model. Susceptible to problems like the vanishing gradient, affecting long-term data analysis.

## Data Availability

Not applicable.
